# Extensive Association of Functionally and Cytotopically Related mRNAs with Puf Family RNA-Binding Proteins in Yeast

**DOI:** 10.1371/journal.pbio.0020079

**Published:** 2004-03-16

**Authors:** André P Gerber, Daniel Herschlag, Patrick O Brown

**Affiliations:** **1**Department of Biochemistry, Stanford University School of MedicineStanford, CaliforniaUnited States of America; **2**Howard Hughes Medical Institute, Stanford University School of MedicineStanford, CaliforniaUnited States of America

## Abstract

Genes encoding RNA-binding proteins are diverse and abundant in eukaryotic genomes. Although some have been shown to have roles in post-transcriptional regulation of the expression of specific genes, few of these proteins have been studied systematically. We have used an affinity tag to isolate each of the five members of the Puf family of RNA-binding proteins in Saccharomyces cerevisiae and DNA microarrays to comprehensively identify the associated mRNAs. Distinct groups of 40–220 different mRNAs with striking common themes in the functions and subcellular localization of the proteins they encode are associated with each of the five Puf proteins: Puf3p binds nearly exclusively to cytoplasmic mRNAs that encode mitochondrial proteins; Puf1p and Puf2p interact preferentially with mRNAs encoding membrane-associated proteins; Puf4p preferentially binds mRNAs encoding nucleolar ribosomal RNA-processing factors; and Puf5p is associated with mRNAs encoding chromatin modifiers and components of the spindle pole body. We identified distinct sequence motifs in the 3′-untranslated regions of the mRNAs bound by Puf3p, Puf4p, and Puf5p. Three-hybrid assays confirmed the role of these motifs in specific RNA–protein interactions in vivo. The results suggest that combinatorial tagging of transcripts by specific RNA-binding proteins may be a general mechanism for coordinated control of the localization, translation, and decay of mRNAs and thus an integral part of the global gene expression program.

## Abstract 2

Messenger RNAs that are associated with each of the five Puf RNA-binding proteins can be grouped according to function and localization, suggesting that tagging of transcripts by specific RNA-binding proteins coordinates the control of their localization, translation, and decay

## Introduction

The dynamic structure and physiology of a cell depend on coordinated synthesis, assembly, and localization of its macromolecular components (Orphanides and Reinberg 2002). The timing and level of expression of the genes that encode these components are controlled by transcription factors that regulate initiation of transcription in a gene-specific manner by binding to specific DNA sequences proximal to the genes they regulate. The combinatorial binding and activity of specific transcription factors confer a distinctive program of regulation on each individual gene while enabling coherent global responses of large sets of genes in physiological and developmental programs. Much less is known about either the system architecture or molecular mechanisms that underlie regulation of the post-transcriptional steps in the gene expression program.

There are approximately 15,000 mRNA molecules in each Saccharomyces cerevisiae cell during exponential growth in rich medium (Hereford and Rosbash 1977) and at least a 10-fold larger number in a typical mammalian cell (Hastie and Bishop 1976). The extent to which the location, activity, and fates of these diverse populations of mRNAs are coordinated and the post-transcriptional mechanisms that might mediate their coordinated regulation remain largely unknown. RNA-binding proteins (RBPs) have been implicated in diverse aspects of post-transcriptional gene regulation, including RNA processing, export, localization, degradation, and translational control (Dreyfuss et al. 2002; Maniatis and Reed 2002; Mazumder et al. 2003). Although there appear to be hundreds of RBPs encoded in eukaryotic genomes (Costanzo et al. 2001; Issel-Tarver et al. 2002), for only a few of these proteins have the RNA targets been systematically identified (Takizawa et al. 2000; Tenenbaum et al. 2000; Brown et al. 2001; Hieronymus and Silver 2003; Li et al. 2003; Shepard et al. 2003; Waggoner and Liebhaber 2003). For example, a recent study in S. cerevisiae found that two nuclear RNA export factors were each associated with large and distinct mRNA populations, and common functional themes were found among the 1,000 or so proteins encoded by each population (Hieronymus and Silver 2003). These observations support a role for RBPs in the coordinated regulation of mRNA subpopulations (Keene and Tenenbaum 2002; Keene 2003).

Systematic identification of the mRNA targets of RBPs can be a powerful approach to understanding the cellular roles of RBPs and the mechanisms by which they might regulate the post-transcriptional lives of mRNAs. We have focused first on the Pumilio–Fem-3-binding factor (FBF) (Puf) proteins from S. cerevisiae, which belong to a structurally related family of cytoplasmic RBPs that are implicated in developmental processes in various eukaryotes (Wickens et al. 2002). Puf proteins are defined by the presence of several (typically eight) consecutive repeats of the Pumilio homology domain (Pum-HD), which confers RNA binding activity (Zamore et al. 1997; Wang et al. 2002a). The Puf proteins characterized to date have been reported to bind to 3′-untranslated region (UTR) sequences encompassing a so-called UGUR tetranucleotide motif and thereby to repress gene expression by affecting mRNA translation or stability. Despite the widespread occurrence of Puf family members, only a few mRNA targets have been identified for these RBPs (Wickens et al. 2002). For example, in *Drosophila*, the PUMILIO protein binds maternal *hunchback* mRNA and, in concert with NANOS protein, represses translation of the mRNA at the posterior pole during early embryogenesis. The Caenorhabditis elegans Puf homologs, called Fem-3-binding factors (FBFs), regulate the switch from spermatogenesis to oogenesis by repressing *fem-3* translation, and they are implicated in the propagation of germline stem cells through binding and inhibition of *gld-1* mRNA expression (Zhang et al. 1997; Crittenden et al. 2002). Less is known about the human homologs: PUMILIO-2 protein interacts with DAZ (deleted in azoospermia) protein and is expressed in embryonic stem cells and germ cells, whereas PUMILIO-1 is almost ubiquitously expressed (Moore et al. 2003).

**Figure 1:**
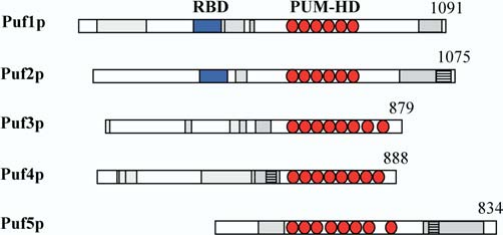
Protein Domain Structure of Yeast Puf Proteins Pum-HD repeats (Zamore et al. 1997) are shown as red ovals and classical RNA-binding domains (RBDs) are depicted as blue boxes. Regions of low complexity, such as proline-, serine-, threonine-, and/or methionine-rich domains, are shown in gray boxes; asparagine stretches are striped. The numbers correspond to the length of proteins in amino acids.

In S. cerevisiae, five proteins, termed Puf1p to Puf5p, bear six to eight Puf repeats (Figure 1). Little is known about the physiological function of these proteins. Mutations in either *PUF4* or *PUF5* result in diminished longevity (Kennedy et al. 1997). *PUF1* was isolated as a multicopy suppressor of certain microtubule mutants (Machin et al. 1995), and a *PUF2* null mutant displayed increased resistance to cycloheximide and paromomycin (Waskiewicz-Staniorowska et al. 1998). However, S. cerevisiae mutants lacking all five *PUF* genes are viable (Olivas and Parker 2000). A genome-wide analysis of mRNA expression patterns in yeast mutants lacking all five *PUF* genes found differential expression of 7%–8% of all mRNAs under steady-state conditions, but no common theme was found among the affected genes (Olivas and Parker 2000). Only two specific mRNA targets have been identified for yeast Puf proteins: Puf3p binds to the *COX17* mRNA 3′-UTR in vitro and may regulate its turnover (Olivas and Parker 2000), and Puf5p negatively regulates expression of reporter genes substituting for the HO endonuclease (Tadauchi et al. 2001).

Using DNA microarrays to identify the specific mRNAs that interact with the five S. cerevisiae Puf proteins, we have found that each Puf protein bound to a large set of distinct and functionally related mRNAs. We identified novel and conserved sequence elements in the mRNAs bound by Puf3p, Puf4p, and Puf5p. The results suggest a system for large-scale coordinated control of cytoplasmic mRNAs and provide insights into the physiological logic of the gene expression program.

## Results

### Systematic Identification of mRNAs Associated with Specific RBPs

**Figure 2:**
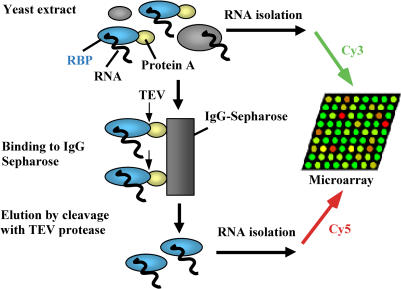
Strategy for Analyzing Genome-Wide RNA–Protein Interactions Protein A-tagged Puf proteins were captured with IgG–Sepharose and released from the beads by cleavage with TEV protease. RNAs associated with the released proteins were isolated, and cDNA copies were fluorescently labeled and hybridized to yeast DNA microarrays. The Cy5/Cy3 fluorescence ratio for each locus reflects its enrichment by affinity for the cognate protein.

To identify RNAs associated with Puf proteins, tandem-affinity purification (TAP)-tagged proteins were purified from whole-cell extracts of S. cerevisiae (Figure 2). The TAP tag (Rigaut et al. 1999), a sequence encoding two IgG-binding units of protein A, a specific protease recognition site, and a calmodulin-binding domain, was fused in-frame at the C-terminus of the respective open reading frame (ORF) in its original chromosomal location (Ghaemmaghami et al. 2003). This design was intended to preserve normal regulation of the expression of the fusion protein. Cells of the TAP-tagged strains showed growth rates and cell morphologies similar to wild-type cells. Cells were grown to mid-log phase in rich medium, extracts were prepared, and ribonucleoprotein complexes were recovered by affinity selection on IgG beads and subsequent cleavage with tobacco etch virus (TEV) protease (see Materials and Methods). To control for nonspecifically enriched mRNAs, the same procedure was performed with wild-type cells lacking the TAP tag. TEV protease cleavage was superior to direct elution of proteins from beads, as it gave lower contamination from nonspecifically interacting RNAs in the resulting purified fractions (data not shown). RNA was isolated from the purified protein samples and from extracts. We obtained 0.8–2 μg of RNA from the Puf affinity-isolated samples gathered from 1-l cultures, but no detectable RNA (<0.1 μg) was recovered when the same procedure was applied to untagged control cells. The yield of RNA from the Puf affinity isolation procedure was sufficient to perform further labeling steps directly, without amplification of RNA by PCR, as had been required in previous studies (Takizawa et al. 2000; Hieronymus and Silver 2003). Two samples from each cell population, total RNA, and RNA isolated by the Puf affinity procedure were used to prepare cDNA probes labeled with different fluorescent dyes, which were mixed and hybridized to S. cerevisiae DNA microarrays containing all known and putative ORFs, introns, and the mitochondrial genome (see Materials and Methods). The ratio of the fluorescent hybridization signals from the two differentially labeled RNA samples, at the array element representing each specific gene, provided an assay for enrichment of the corresponding mRNA by the Puf-affinity procedure.

**Figure 3:**
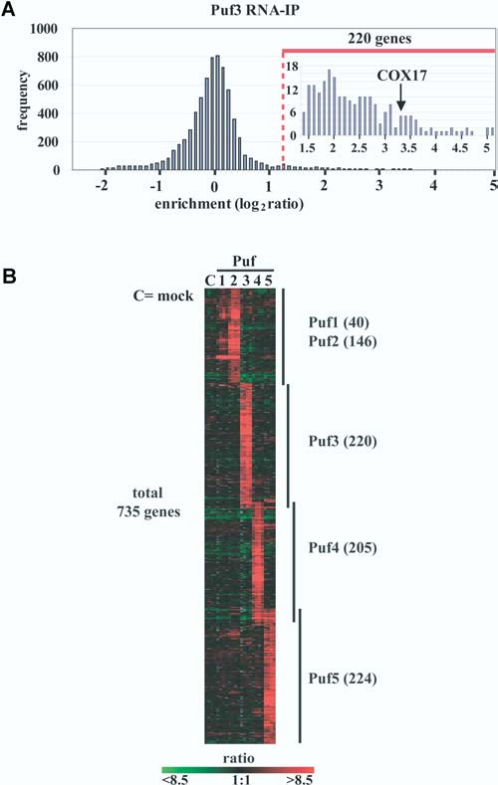
Defining Puf Target RNAs (A) Distribution of average Cy5/Cy3 fluorescence ratios from four independent microarray hybridizations analyzing Puf3p targets. The arrow depicts enrichment of *COX17* mRNA, which is known to bind to Puf3p (Olivas and Parker 2000). The red dashed line indicates the threshold applied for defining 220 target RNAs (a magnification is shown of the enriched region). (B) Cluster of RNA targets for Puf proteins. Rows represent genes (unique cDNA elements) and columns represent individual experimental samples. Each Puf protein and an untagged strain (mock control) were assayed in quadruplicate. The color code indicates enrichments (green–red color scale). The number of mRNAs interacting with each Puf protein is indicated in parentheses. mRNAs clustering with the mock controls were removed as false positives (see Materials and Methods).

Puf3p is the only one of the five S. cerevisiae Puf proteins for which direct in vitro interaction with an mRNA (*COX17*) has previously been described, thereby providing an internal positive control (Olivas and Parker 2000). *COX17* mRNA was substantially and consistently enriched in four independent Puf3p affinity isolations (ratio = 10 ± 1.4; Figure 3A), but not in mock isolations (ratio = 0.8 ± 1.2). In general, after filtering for spots with high background or irregular shapes, enrichment values for the entire set of arrayed sequences were reproducible (median of standard deviations in all arrayed spots = 0.35 on a log_2_ scale) (see Materials and Methods). To define targets specific to each Puf protein, we first selected all sequences for which enrichment factors in the corresponding affinity isolation procedures were at least two standard deviations above the mean for all arrayed sequences (Figure S1; for samples isolated by the Puf3p-affinity procedure, this corresponded to an enrichment factor of greater than or equal to 2.5). Second, we eliminated from this selected group any sequences that were also consistently enriched in the mock procedure (see Materials and Methods). Although no cutoff can perfectly distinguish the actual physiological targets from false positives, the high reproducibility of the results (see Figure 3B), the occurrence of distinct mRNA populations associated with the different Puf proteins, and the characterization of these targets described in the subsequent sections, including the identification of distinct sequence motifs and in vivo confirmation of the role of these motifs in specific RNA–protein interactions, strongly support the validity of the majority of the targets. Finally, the list of target mRNAs did not change substantially by application of other statistical methods for selection (see Lieb et al. 2001).

A large number of arrayed sequences, 818, identified transcripts associated with at least one Puf protein (see Figure 3B; Table S1), with 735 encoding distinct ORFs. This represents approximately 12% of the known and predicted protein-coding sequences in the S. cerevisiae genome. Of these, 90 transcripts interact with more than one Puf protein. The largest overlap was observed between the groups of transcripts associated with Puf1p and Puf2p—which also have the greatest overall similarity in amino acid sequence among the Puf proteins (45% identical); 36 of the 40 Puf1p targets were also associated with Puf2p. Twenty-eight mRNAs were bound by both Puf4p and Puf5p, and 16 were bound both by Puf2p and Puf5p. Seven transcripts were enriched with three different Puf proteins (*DHH1* and *YOL109w* mRNAs with Puf1p, Puf2p, and Puf5p; *NOP1* mRNA with Puf1p, Puf4p, and Puf5p; *SUR7* and *SFL1* mRNAs with Puf2p, Puf4p, and Puf5p; and *IFM1* mRNA with Puf3p, Puf4p, and Puf5p). The remaining 645 target mRNAs were each associated with only one of the Puf proteins. Thus, each Puf protein associates with a distinct and highly specific subset of mRNAs (see Tables S3–S7).

We estimated the number of Puf proteins per cell by a filter affinity blot analysis using protein A as a standard for calibration (Table S2). We found that Puf1p, Puf2p, Puf3p, and Puf5p were similar in abundance, with 350–400 molecules per cell. Puf4p was approximately twice as abundant (approximately 900 molecules per cell). The relatively low abundance of the Puf proteins is therefore comparable to that of transcription factors, protein kinases, and cell cycle proteins (Futcher et al. 1999). Moreover, our measurements imply that the intracellular concentrations of the Puf proteins range between 20 and 50 nM, approximately one order of magnitude higher than the dissociation constants for binding of their metazoan homologs to the cognate target RNAs. The number of Puf proteins per cell approximates the estimated numbers of cognate Puf target mRNA molecules present in the cell (Holstege et al. 1998; Wang et al. 2002b) (Table S2), consistent with a model in which each Puf protein molecule is associated with one mRNA molecule in the cell.

### Puf3p Specifically Binds mRNAs Encoding Mitochondrial Proteins

As a first step toward identifying functional themes among the mRNAs associated with each Puf protein, we retrieved the Gene Ontology (GO) annotations for process, function, and compartment from the *Saccharomyces* Genome Database (SGD) (Issel-Tarver et al. 2002). (The target mRNAs for each Puf protein are listed in Tables S3–S7.) We then searched for significant shared GO terms in the lists of Puf mRNA targets (Table S8).

**Figure 4:**
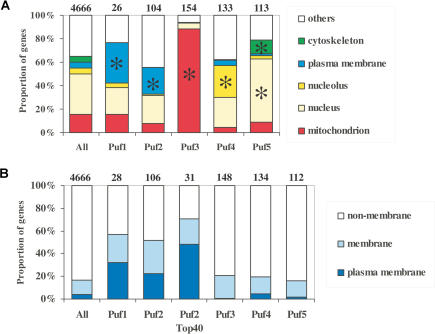
Classification of mRNAs Interacting with Puf Proteins (A) Column charts showing compartmentalization of characterized gene products encoded by the Puf targets. The same compartments are shown for the entire genome in the columns designed “All” (YPD, May 2003). The number of genes represented in the charts is indicated on the top of columns. An asterisk indicates classes with *p* values of less than 0.001. (B) Fraction of membrane-associated gene products among the Puf targets. We classified the targets by combining both GO and YPD annotations (May 2003). “Plasma membrane” (light blue) is a subpopulation of the total membrane-associated proteins (blue). Soluble cytoplasmic or nuclear proteins were classified as “non-membrane.” “All” refers to the genome-wide compartmentalization of characterized genes, and respective numbers were retrieved from YPD. “Puf2 Top 40” refers to the 40 highest enriched Puf2p targets and equals the total number of Puf1p targets.

Puf3p associated almost exclusively with transcripts of nuclear genes that encode mitochondrial proteins (*p* < 10^−88^; see Table S5). In particular, of the 154 Puf3p-associated transcripts for which GO annotation of subcellular localization was available, 135 (87%) were assigned to mitochondria (Figure 4A). Of the Puf3p-associated mitochondrial gene products, 80 (59%) are involved in protein biosynthesis, including structural components of the ribosome (55 genes), tRNA ligases (12 genes), and translational regulators (nine genes). Twenty-two of the Puf3p-bound transcripts are involved in mitochondrial organization and biogenesis, 17 in aerobic respiration, and 12 in mitochondrial translocation. Based on this striking cytotopic (relating to location in the cell) concordance, we suggest that the remaining 66 Puf3p mRNA substrates (30%) for which no GO annotations were available are likely to encode mitochondrial proteins. (While this paper was under review, a genome-wide analysis of protein localization in S. cerevisiae [Huh et al. 2003] reported a mitochondrial localization for 27 additional Puf3p targets, raising the total to 162 of the 220 putative Puf3p mRNA targets encoding mitochondrial proteins.)

### Puf1p- and Puf2p-Associated mRNAs Disproportionately Encode Membrane-Associated Proteins

Of all the characterized S. cerevisiae genes for which any information about subcellular localization is available, 18% are currently classified as encoding membrane-associated proteins (Yeast Proteome Database [YPD], May 2003; see Costanzo et al. 2001). A much greater fraction of the mRNAs associated with Puf1p and Puf2p encode membrane-associated proteins: 16 of the 28 (57%) known proteins encoded by Puf1p-interacting mRNAs and 55 of 106 (52%) known proteins encoded by Puf2p-interacting mRNAs (see Figure 3B; see Tables S3 and S4). Transcripts encoding proteins associated with the plasma membrane were particularly enriched among the Puf1p- and Puf2p-bound mRNAs. Most of the mRNAs bound by Puf1p were also associated with Puf2p. However, Puf2p bound uniquely to many additional mRNAs (146 Puf2p mRNA targets versus 40 for Puf1p). In terms of cellular processes, many Puf1p- and Puf2p-associated transcripts encode proteins with roles in transmembrane transport and vesicular trafficking of proteins: 9 out of 26 (34%; *p* < 0.0002) of annotated Puf1p targets and 24 out of 104 (23%; *p* < 10^−5^) annotated Puf2p targets (compared to 9% of all characterized genes) (YPD, May 2003). This group includes transporters for spermine (Tpo1, Tpo2, Tpo3), proteins (Nce101, Nce102, Ast1, Vps72, Mas6, Sfk1, Mup3), vesicles (Sso2, Snc2, Yip1, Aps3, Ypr157w), and lipids (Pdr16, Ykl091c, Fps1 [glycerol]). (Tpo2 and Tpo3 may cross-hybridize on arrays because of their high sequence identity [89%], but Tpo1 does not [Shepard et al. 2003]).

### Puf4p and Puf5p Interact Selectively with mRNAs Encoding Nuclear Components 

Among the Puf5p targets (see Table S6), we found two common themes. First, a remarkable fraction encodes nuclear proteins that participate in covalent modification of histones, chromatin-remodeling complexes, or transcriptional regulation (64 of the 113 annotated genes [57%; *p* < 3 × 10^−6^]). Second, the Puf5p-associated transcripts included a substantial fraction of the mRNAs known to encode components or regulators of the mitotic spindle apparatus in yeast: 14 mRNAs that encode microtubule-based spindle components, including seven of the 25 (28%; *p* < 4 × 10^−5^) structural components of the spindle pole body (Kar1, Ccd31, Spc19, Spc42, Bbp1, Cnm67, and Nuf2) (Wigge et al. 1998). Messages encoding nuclear and cytoplasmic proteins that regulate polarized growth (Ame1, Boi2, Bsp1, Bub1, Bud9, Dad2, Elm1, Gic1, Kar9, Rax2, Ste7), some of them known to interact with spindle components, were also Puf5p targets.

Transcripts encoding nucleolar proteins were highly enriched among the Puf4p-bound mRNAs: 36 of the 133 (27%) annotated genes in this group encode nucleolar proteins, as compared to 3% of all the annotated genes in the S. cerevisiae genome (*p* < 10^−12^). Of these 36, 29 are directly involved in ribosomal RNA (rRNA) synthesis, processing, and ribosome maturation (*p* < 10^−15^), major functions of the nucleolus (Fatica and Tollervey 2002; Gerbi et al. 2003) (see Tables S5 and S8).

Twenty-eight transcripts were enriched in both the Puf4p and Puf5p affinity isolations, including six transcripts encoding components of the nucleosome (*p* < 10^−11^), among them the four core histone proteins (histones 2A and 2B, histone 3, and histone 4; note that histones 2A and 2B are 98% identical and therefore cross-hybridize).

### Diverse Functional Links among Transcripts Associated with Each Puf Protein

In addition to the cytotopic relationships within each group of Puf-associated mRNAs, we were struck by the frequency with which transcripts encoding different components of protein complexes or systems of interacting proteins were bound by the Puf proteins. For example, most of the nuclear transcripts encoding components of the mitochondrial ribosome (55 out of the 77 known genes; Gan et al. 2002) were Puf3p-associated. This observation prompted us to search for other protein complexes and functional systems that shared similarly Puf-associated mRNAs.

Other examples of coordinate “tagging” of transcripts encoding subunits of multiprotein complexes include Puf4p association of mRNAs encoding three of the four protein components of the H/ACA core particle (Cbf5p, Gar1p, and Nhp2p), which synthesizes pseudouridine in rRNAs (Henras et al. 1998) (Figure S2; no data were obtained for the fourth component, Nop10p). Puf5p bound mRNAs encoding histone acetylases (Ada2p, Spt8p, and Hfi1p), which are components of the Spt–Ada–Gcn5–acetyltransferase (SAGA) complex, and transcripts encoding at least four of the six members of the RSC (remodels the structure of chromatin) family of DNA-stimulated ATPases with bromodomains (Bdf1p, Bdf2p, Rsc2p, and Rsc4p; no array data were obtained for the two other members, Rsc1p and Spt7p). As mentioned above, the mRNAs encoding at least three of the four core histones were enriched in both Puf4p and Puf5p affinity isolations.

We also found numerous cases in which the transcripts encoding multiple members of a functional group of proteins were bound by the same Puf protein. For example, the transcripts encoding the Tpo1, Tpo2, and Tpo3 proteins, the three known spermine transporters in the plasma membrane (Albertsen et al. 2003; see note above about cross-hybridization), and the two known genes implicated in the nonclassical protein export pathway (*NCE101*, *NCE102*) (Cleves et al. 1996) were bound by Puf1p and Puf2p and by Puf2p, respectively. Puf5p was associated with all of the histone deacetylases (HDACs) that act on histones located around coding sequences—Sin3p (a class I HDAC), Hda1p (a class II HDAC), and both components of the Set3C complex (Hst1p and Snt1p) (Kurdistani and Grunstein 2003). (Two other HDACs, Hos1p and Hos3p, which deacetylate histones around the ribosomal DNA locus, were not enriched in Puf5p affinity isolations.)

Finally, we identified cases in which the mRNAs encoding multiple components of a specific regulatory system were bound by the same Puf protein. For example, Puf2p associates with mRNAs encoding diverse proteins regulating Pma1p, which is an ATP-dependent proton transporter located in the plasma membrane, and with *PMA1* mRNA itself (Figure S2). All of the mRNAs encoding nucleolar glycine/arginine-rich (GAR) domain-bearing proteins (Sbp1p, Nsr1p, Nop1p, Gar1p) as well as *HMT1* mRNA, encoding a dimethylase that modifies the nucleolar GAR proteins (Xu et al. 2003), were associated with Puf4p, while none of the mRNAs encoding the distinct group of nonnucleolar GAR proteins were bound by Puf4p (Figure S2).

### Sequence Motifs in the 3′-UTR of mRNA Targets Direct Binding by Puf Proteins

**Figure 5:**
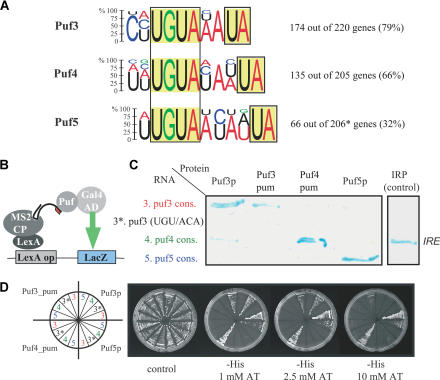
Sequence Motifs Interacting with Puf Proteins (A) Consensus motifs detected within 3′-UTR sequences of Puf3p, Puf4p, and Puf5p target mRNAs. Height of the letters specifies the probability of appearing at the position in the motif. Letters with less than 10% appearance were omitted. Fraction of genes bearing a motif in the 3′-UTR sequence is indicated to the right. Y-helicase proteins are nearly identical in sequence and were excluded from this analysis. (B) Scheme of three-hybrid assay for monitoring RNA–protein interactions in vivo (Bernstein et al. 2002). (C) β-Galactosidase activity for three-hybrid assay. Proteins assayed are indicated on top, RNAs to the left. Abbreviations: pum, pum-HD; cons., consensus motif; UGU/AGA, UGU in consensus sequence mutated to AGA. (D) Activation of *HIS3* reporter gene and resistance to 3-aminotriazole (3-AT), a competitive inhibitor of the *HIS3* gene product, in a three-hybrid assay (Bernstein et al. 2002).

The Puf homologs in *Drosophila* and C. elegans bind to sequences in the 3′-UTR of mRNAs (Wickens et al. 2002). We therefore examined the sets of mRNAs associated with each of the S. cerevisiae Puf proteins for the presence of common sequence motifs in 5′-UTRs and 3′-UTRs, using multiple expectation maximization for motif elicitation (MEME) as a motif discovery tool (Bailey and Elkan 1994). We identified distinct 10- or 11-nucleotide sequence motifs in the 3′-UTR among the mRNAs interacting with Puf3p, Puf4p, and Puf5p (Figure 5A, Tables S9–S11). We have thus far been unable to identify conserved sequence elements among Puf1p and Puf2p targets; these proteins may recognize structural elements in the RNA rather than simple sequence strings, possibly via their classical RNA-binding domains instead of their six-repeat Pumillio domains.

The conserved motifs we identified in the Puf3p, Puf4p, and Puf5p targets each include a UGUR tetranucleotide sequence, which is a feature of all previously reported RNA targets of Puf family proteins (Wickens et al. 2002). Furthermore, in each case, the consensus sequence contains a conserved dinucleotide (UA), located two, three, or four nucleotides downstream of the UGUR motif, in the consensus sites for Puf3p, Puf4p, and Puf5p. Remarkably, the Puf3p consensus motif matches a sequence (CYUGUAAAUA) previously identified by computational tools in 3′-UTR sequences of nuclear genes coding for mitochondrial proteins (Jacobs Anderson and Parker 2000).

We examined the distribution of the consensus sequence motifs in the entire S. cerevisiae genome (Table 1). Of the genes whose mRNAs were predicted by computational analysis to contain one of these three target sequences in their 3′-UTRs, 42% were identified experimentally as targets in the corresponding affinity isolation procedure (Table 1). The consensus motifs were occasionally found in the coding sequence of an experimentally identified target gene, but were much rarer in the predicted 5′-UTR sequences (Table 1). Moreover, only a few mRNAs had two copies of the motifs: five mRNAs among the Puf3p targets, six among the Puf4p targets, and one among the Puf5p targets (see Tables S5–S7). As our computational method did not detect the cognate consensus sequence elements in all the experimentally identified targets, alternative sequences or structural elements in RNAs might also allow specific interactions with Puf proteins, some mRNAs may be associated indirectly as part of larger complexes, and some of the putative mRNA targets identified by our affinity procedure are likely to be false positives.

**Table 1:**
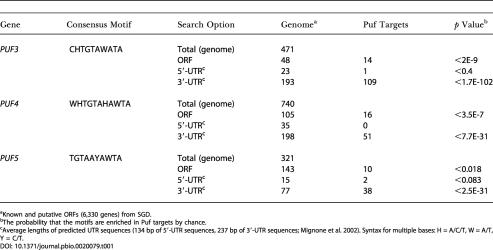
Number of Consensus Motifs Found in the Genome and in Puf Targets

^a^Known and putative ORFs (6,330 genes) from SGD

^b^The probability that the motifs are enriched in Puf targets by chance

^c^Average lengths of predicted UTR sequences (134 bp of 5′-UTR sequences, 237 bp of 3′-UTR sequences; Mignone et al. 2002). Syntax for multiple bases: H = A/C/T, W = A/T, Y = C/T

To test the in vivo function of the putative recognition elements identified by the computational analysis, we assayed RNA–protein interactions in vivo using the yeast three-hybrid system (Bernstein et al. 2002) (see Figure 5B). Puf3p, Puf4p, and Puf5p bound specifically to a sequence matching to the cognate consensus sequence, as assayed by activation of the *lacZ* and *HIS3* reporter genes (see Figure 5C and 5D). For Puf3p and Puf4p, the Pum-HD alone was sufficient to confer specific binding (see Figure 5C and 5D), but no interaction could be seen with the Puf5p Pum-HD alone (data not shown). These interactions were specific: mutations in the UGU of the Puf3p consensus sequence disrupted binding, and each Puf protein interacted with its cognate consensus sequence in preference to the closely related consensus sequences recognized by the other Puf proteins. We detected a weak interaction between Puf3p and the Puf4p target sequence, an interaction that was not seen with the Puf3p Pum-HD alone. These results suggest that binding of the Puf proteins to these specific *cis*-acting elements directs their functions to specific sets of mRNAs.

### Subcellular Distribution of Puf Proteins

**Figure 6:**
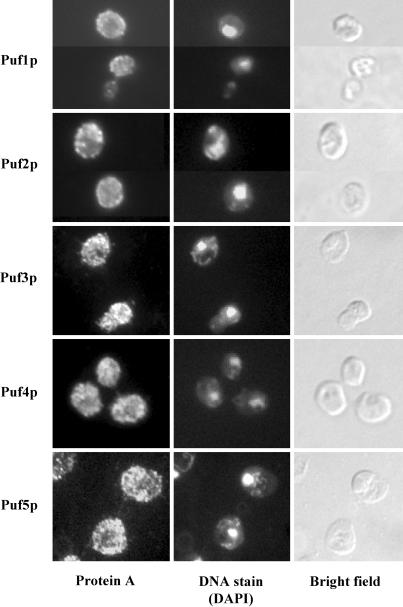
Localization of Puf Proteins TAP-tagged Puf proteins were visualized in fixed cells. DNA was costained with 4′,6-diamidino-2-phenylindole dimethylsulfoxide (DAPI).

We investigated the localization of the TAP-tagged Puf proteins by immunofluorescence with antibodies against the TAP tag (see Materials and Methods). All five Puf proteins were predominantly localized to multiple discrete foci in the cytoplasm (Figure 6). The predominantly cytoplasmic localization is consistent with previous reports for S. cerevisiae Puf3p and Puf5p (Tadauchi et al. 2001) and for the homologous proteins in higher eukaryotes (Lehmann and Nüsslein-Volhard 1991; Zhang et al. 1997). The distribution of the foci of Puf proteins was not obviously related to distinct cellular organelles or structures, with the exception of Puf1p and Puf2p, which localized in foci enriched near the periphery of the cell. Because of the diffuse and pleiomorphic distribution of mitochondria in the cell, we cannot exclude the possibility that Puf3p, which specifically bound transcripts of proteins destined for the mitochondria, is associated with mitochondria.

### Altered Levels of Puf3p-Associated mRNAs in a *puf3*Δ Mutant 

**Figure 7:**
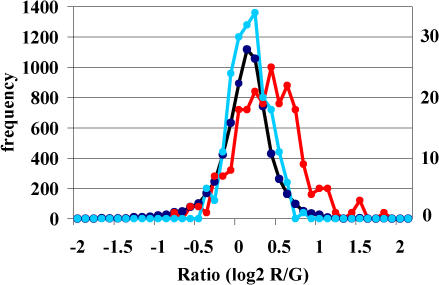
Gene Expression Profiling of *puf3* Mutants Distribution of average Cy5/Cy3 fluorescence ratios from three independent microarray hybridizations comparing mRNA levels of *puf3*Δ with wild-type cells grown in minimal media with glycerol. The left frequency axis refers to all genes (black line); the axis to the right refers to Puf3p and Puf4p (control) targets, shown as red and blue lines, respectively. Relative expression levels of the 220 Puf3p mRNA targets in *puf3*Δ cells were selectively increased compared to all other mRNAs analyzed (*p* < 10^−34^), whereas Puf4p targets were not (*p* > 0.05). Thirty-nine genes involved in aerobic respiration (according to GO annotation and SGD), but not bound by Puf3p, were similarly enriched (*p* < 5 × 10^−5^) in the *puf3* mutant as random sets of 39 Puf3p targets (*p* < 10^−6^). Likewise, 220 randomly selected mRNAs coding for mitochondrial proteins that were not associated with Puf3p in the experiments herein were weakly enriched in the mutant (*p* < 10^−8^).

A previous study compared steady-state mRNAs levels of cells bearing deletions of all five Puf proteins and wild-type cells grown in rich media (Olivas and Parker 2000). Only 12 of the 148 (8%) mRNAs whose abundance changed by more than 2-fold were selectively enriched in our affinity isolations with Puf proteins. The lack of a simple relationship between the mRNA binding specificity we observed and the reported effects of these multiple mutations on global gene expression prompted us to design a more specific experiment to search for a possible connection between specific mRNAs levels and binding to Puf proteins. We focused on Puf3p, as its strong association with mRNA-encoding mitochondrial proteins suggested that we should look for a regulatory function for this protein in mitochondrial physiology. Indeed, we found that *puf3*Δ cells grew more slowly than isogenic *puf3^+^* cells on minimal media plates with glycerol as the carbon source (Figure S3). We therefore compared mRNA levels in the *puf3*Δ and *puf3^+^* cells grown under these conditions by DNA microarray hybridization. Although the magnitude of the change was small, the relative expression levels of the 220 Puf3p-associated mRNAs were selectively increased in *puf3*Δ cells, compared to all other mRNAs analyzed (*p* < 10^−34^) (Figure 7). Of the 16 mRNAs whose abundance was increased by more than 2-fold in the *puf3*Δ mutant, 11 (70%) were among the transcripts identified as Puf3p targets by our co-purification experiments, and all encode mitochondrial proteins. This result could reflect a direct effect of Puf3p on its target mRNAs, for example, by promoting mRNA decay (Olivas and Parker 2000). However, the levels of transcripts involved in respiration and mitochondrial function, including many that did not appear to be bound directly by Puf3p, were increased in the *puf3*Δ mutant, suggesting the possibility that the elevated abundance of Puf3p target mRNAs could instead be an indirect response to impaired mitochondrial and respiratorial function in *puf3*Δ cells.

## Discussion

In an analysis of just five of the hundreds of RBPs encoded by the S. cerevisiae genome, we found that more than 700 transcripts appeared to be specifically bound by one or more RBPs, with each of the five Puf family proteins “tagging” a distinct set of mRNAs. These sets encode functionally and cytotopically related proteins. For three of the Puf proteins, we identified distinct short sequences in the associated specific set of mRNAs, typically in the 3′-UTR, which were sufficient for specific binding to the cognate Puf protein in vivo. Many sets of mRNAs encoding proteins localized to the same subcellular compartment, protein complex, or functional system were bound by the same Puf protein. Puf3p, which specifically associated with cytoplasmic mRNAs encoding mitochondrial proteins, generally affected the steady-state levels of its mRNA targets as reflected by their increased abundance in *puf3* mutant cells.

The selective “tagging” by sequence-specific RBPs of mRNAs that share common physiological roles suggests a general and widespread mechanism for coordinated control of their expression. Previous reports have identified coordinated regulation of small sets of functionally related mRNAs by specific RBPs. For example, mammalian stem–loop binding protein (SLBP) associates with all five classes of histone mRNAs and guides proper 3′-end formation (Dominski and Marzluff 1999). Iron regulatory proteins (IRPs) bind to and regulate translation of five different mRNAs encoding proteins involved in iron metabolism (Eisenstein and Ross 2003), and a cytoplasmic poly(A) polymerase regulates multiple mRNAs in early development (Mendez and Richter 2001). Based on these and other examples (Tenenbaum et al. 2000), Keene and Tenenbaum (2002) have suggested that messenger RBPs could define “post-transcriptional operons.” Our results provide strong support for this general idea of coordination of gene expression via RBPs and suggest that the post-transcriptional control afforded by combinatorial binding of RBPs to mRNAs could allow greater regulatory flexibility than a simple operon (see also Keene and Tenenbaum 2002). Further, we suggest that RBPs may play important roles in subcellular localization and efficient assembly of protein complexes.

**Figure 8:**
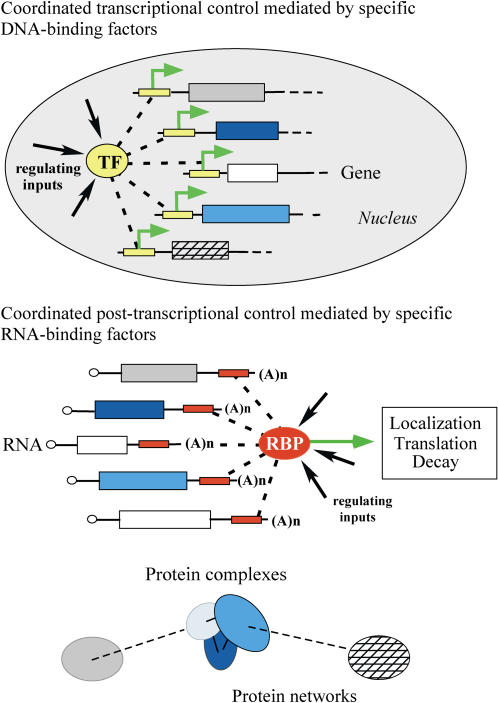
Specific Proteins Bind Functional Groups of Genes for Regulation At the transcriptional level (top), transcription factors (TFs) regulate initiation of transcription (green arrow) in the nucleus by binding to sequence elements (yellow box) proximal to their target coding regions (boxes). At the post-transcriptional level (middle), RBPs regulate decay, translation, or localization of mRNAs in a coordinated fashion by interaction with sequence/structural elements in the RNA that are often found in 3′-UTR regions (red box). Functional relations at the protein level (bottom) can be reflected at both the transcriptional and post-transcriptional levels: sets of genes that encode functionally related proteins, such as subunits of stochiometric complexes (blue) or components of the same regulatory or metabolic pathway (gray and cross-hatched boxes), may be regulated by common transcription factors and their mRNAs post-transcriptionally coregulated by RBPs (dashed interactions).

The RBPs encoded in eukaryotic genomes rival specific transcription factors in their numbers and diversity, raising the intriguing possibility that specific regulation of the localization, translation, and survival of mRNAs might be comparable in their richness and complexity to regulation of transcription itself. Each of the five Puf proteins interacts with a distinct large set of mRNAs, comprising more than 700 different mRNAs in total. Five other RBPs in S. cerevisiae have been subjected to a similar genome-wide survey of their mRNA targets. She2p, which plays a critical role in selective targeting of specific mRNAs to the bud tip (Shepard et al. 2003), Khd1p, which has also been implicated in localizing gene expression to the nascent bud (A. P. Gerber, unpublished data), and Scp160p, an RBP implicated in genome stability (Li et al. 2003), were each found to bind from 20 to hundreds of distinct mRNAs, and two proteins implicated in RNA export from the nucleus, Yra1p and Mex67p, were each associated with more than 1,000 mRNAs (Hieronymus and Silver 2003). Thus, just ten of the 567 S. cerevisiae proteins known or predicted from the genome sequence to have RNA binding activity (Costanzo et al. 2001) have been found to bind, in a functionally specific pattern, a total of approximately 2,500 different transcripts (approximately 40% of the transcriptome). The extent and specificity of the RNA–protein interactions represented by the proteins studied to date, extrapolated to the hundreds of putative RBPs that remain to be investigated, suggest the existence of an extensive network of RNA–protein interactions that coordinate the post-transcriptional fate of large sets of cytotopically and functionally related RNAs through each stage of its “lifecycle.” It further suggests a potential regulatory repertoire comparable in its diversity and richness to that of the DNA-binding transcription factors (Figure 8). Indeed, the combinatorial binding of mRNAs by multiple RBPs could, in principle, define a specific post-transcriptional fate for each individual mRNA (for an example, see Sonoda and Wharton 2001).

Many sets of mRNAs bound by the same Puf protein encode proteins that act in the same subcellular location, form stochiometric complexes, or are implicated in the same cellular pathway. This organization is most clearly exemplified by Puf3p, which selectively bound mRNAs encoding mitochondrial proteins, including at least 70% of all mitochondrial ribosomal proteins (see Figure 4). Combinations of RBPs could specify smaller sets of RNAs encoding more precisely defined functional groups of proteins. For example, the mRNAs encoding the core histone proteins were among the small set of mRNAs that were associated with both Puf4p and Puf5p. These results therefore hint that networks of functional and physical interactions among proteins could be reflected in a corresponding network of mRNA–protein interactions that coordinate post-transcriptional control of their expression and fate.

For three of the Puf proteins, we found that RNA–protein interactions were directed by compact sequence elements, usually located in the 3′-UTR of the mRNA (see Figure 5). Interactions with 3′-UTR sequences have been described for many cytoplasmic RBPs involved in post-transcriptional regulation (Mazumder et al. 2003). Our analysis has revealed that such recognition elements are probably much more widespread than previously recognized. Sequence and structural elements in mRNAs that are related to the function or cellular localization of the encoded proteins may be a general feature of eukaryotic genes, paralleling the role of the DNA sequences that direct specific transcription factors to promoters and enhancers (Cliften et al. 2003).

The multifocal cytoplasmic distribution of Puf proteins raises the possibility that the mRNAs associated with each Puf protein are colocalized (see Figure 6). In mammalian cells, specific mRNA molecules and specific messenger RBPs have also been found to be localized to specific “granular” subcytoplasmic loci, although the generality of this phenomenon has not been established (Andersen and Kedersha 2002; Eystathioy et al. 2002; Farina et al. 2003). One function of the Puf proteins and related proteins that bind specific families of mRNAs could be to localize functionally related mRNAs to specific cytoplasmic loci. Physical clustering of functionally related groups of mRNAs could aid the assembly of complexes and the coordinated control of translation or RNA turnover. In support of this idea, it has recently been suggested that mRNA decay in the cytoplasm of S. cerevisiae occurs in distinct loci (Sheth and Parker 2003) and, further, that mRNAs encoding different subunits of stoichiometric complexes do indeed have concordant decay rates (Wang et al. 2002b). We propose that the location in the cell at which any mRNA is translated or degraded is not left to chance. Instead, every mRNA that leaves the nucleus may be delivered, in a process directed by specific protein–RNA interactions, to one of a limited number of specific foci in the cytoplasm, designated as destinations for a specific functionally related family of mRNAs. These foci could serve to colocalize and coregulate synthesis of proteins that need to assemble or act together, thereby facilitating efficient and rapid assembly and localization of the proteins. The number of distinct families of functionally specialized foci may be quite large. The locations of these foci need not correspond to recognizable cellular features, but may simply be ad hoc sites for localized, coordinated translation of proteins that are to be assembled into a complex or a functional unit. Specific predictions of this hypothesis, such as colocalized translation of the subunits of stoichiometric complexes, should be amenable to direct experimental tests.

Combinatorial binding of mRNAs by specific regulatory proteins, linking their post-transcriptional regulation to specific signal transduction pathways, could allow rapid and efficient reprogramming of gene expression during development or in response to changing physiological conditions. Indeed, regulation of specific genes by external signals via RPBs has been described in higher eukaryotes (Lasko 2003). For example, the signal transduction and activation of RNA (STAR) proteins contain RNA-binding motifs combined with protein–protein interaction domains and phosphorylation sites, which could allow integration of stimuli conducted by signal transduction cascades (Lasko 2003). Similarly, the Puf proteins contain numerous putative phosphorylation motifs, as well as domains with characteristics often implicated in protein–protein interactions, such as glutamine/arginine-rich regions (Michelitsch and Weissman 2000) (see Figure 1).

Coordination of cellular processes has long been thought to be mediated primarily at the transcriptional and post-translational level. Our results join a growing body of studies (Tenenbaum et al. 2000; Eystathioy et al. 2002; Wang et al. 2002b; Hieronymus and Silver 2003; Shepard et al. 2003; see also Keene and Tenenbaum 2002) that suggest that the localization, translation, and stability of mRNAs are subject to extensive and important regulation and coordination by interaction with a diverse set of RBPs. Systematic mapping of these interactions and deciphering their roles, molecular mechanisms, and coordination will undoubtedly yield important new insights into biological regulation and the gene expression program.

## Materials and Methods

### 

#### Oligonucleotide primers

Restriction sites are in italics: Puf3-F1, 5′-cg*ggatcc*ATGGAAATGAACATGGATATGGATATGG-3′; Puf3-R1, 5′-g*gaattc*TCACACCTCCGCATTTTCAACCAATG-3′; Puf3-F6nco, 5′-*cCATGg*CACTAAAAGACATCTTTGG-3′; Puf4-F2nco, 5′-*ccatgG*CGGACGCAGTTTTAGACCAATA-3′; Puf4-R1eco, 5′-*gaattc*gTGAATCTAAATGTAACATTCCG-3′; Puf5-F2nco, 5′-*ccATGG*TCGAAATCAGCGCACTACC-3′; Puf5-R1xho, 5′-*ctcgag*cACTTGGAAGTAATTCTTTTGTA-3′; M16-1, 5′-GGG*CTCGAG*tagggaataccttgtaaatatcctatgaaaGCATG-3′; M16-2, 5′-Ctttcataggatatttacaaggtattcccta*CTCGAG*CCC-3′; M16-1mut, 5′-GGG*CTCGAG*tagggaatacctacaaaatatcctatgaaaGCATG-3′; M16-2mut, 5′-Ctttcataggatattttgtaggtattcccta*CTCGAG*CCC-3′; Caf-1, 5′-GGG*CTCGAG*tgggcacgattgtaataatacttcatgataaGCATG-3′; Caf-2, 5′-Cttatcatgaagtattattacaatcgtgccca*CTCGAG*CCC-3′; Yor-1, 5′-GGG*CTCGAG*gctttcatcatctgtataatatttatatgtcGCATG-3′; and Yor-2, 5′-Cgacatataaatattatacagatgatgaaagc*CTCGAG*CCC-3′.

#### Strains and plasmid construction

The TAP-tagged Puf3p strain (SC1249) was obtained from Cellzome (Heidelberg, Germany) (Gavin et al. 2002). TAP-tagged Puf1p, Puf2p, Puf4p, and Puf5p strains were a gift from Dr. Erin O'Shea (Ghaemmaghami et al. 2003). Correct genomic integration of each tag was verified by PCR and by immunoblot analysis of cell extracts (data not shown). Strain BY4741 was used for mock-control affinity isolations of RNA, and deletions of the *PUF3* and *PUF4* genes in this strain were obtained from Dr. Ron Davis (Winzeler et al. 1999).

The ORF of *PUF3* was amplified by PCR with primers Puf3-F1 and Puf3-R1 from S. cerevisiae genomic DNA and cloned into pCR2.1 using the TOPO TA Cloning Kit (Invitrogen, San Diego, California, United States). The *PUF3* ORF was sequenced and subcloned into pACTII via NcoI and EcoRI restriction sites, resulting in plasmid pACTII-Puf3. A full-length Puf5p construct pGAD-*MPT5* was a gift from Dr. Kenji Irie (Tadauchi et al. 2001).

Sequences encoding the Pum-HD domains of Puf3p (amino acids 535–879), Puf4p (amino acids 557–888), and Puf5p (amino acids 202–578) were PCR-amplified from genomic DNA with oligo pairs Puf3-F6nco/Puf3-R1, Puf4-F2nco/Puf4-R1eco, and Puf5-F2nco/Puf5-R1xho, respectively. Products were ligated into pCR2.1-TOPO, sequenced, and further cloned into pACTII via restriction sites present in the oligonucleotides used for amplification.

The RNA consensus sequences interacting with Puf proteins plus ten nucleotides of flanking sequences were cloned into the SmaI and SphI sites of the vector pIIIA/MS2-2 (Bernstein et al. 2002) using annealed synthetic oligonucleotides. The *PUF3* RNA consensus sequence spanning nucleotides 24–33 in the 3′-UTR of YBL038w/*MRPL16* was constructed with oligonucleotides M16-1 and M16-2. In M16mut the conserved UGU motif was changed to ACA. The *PUF4* consensus (nucleotides 24–34 in the 3′-UTR of YOR145c) was constructed with oligonucleotides Yor-1 and Yor-2. The *PUF5* consensus (nucleotides 105–114 in the 3′-UTR of YNL278w/*CAF120*) was constructed with oligonucleotides Caf-1 and Caf-2.

#### Isolating RNAs specifically associated with selected RBPs

For a detailed protocol, see the Supporting Information on our Web site. In brief, 1 l of cells were cultured in YPAD medium (yeast–peptone–dextrose [YPD] supplemented with 20 mg/ml adenine–sulfate) at 30°C and collected during exponential growth by centrifugation. Cells were washed twice with ice-cold buffer A (20 mM Tris–HCl [pH 8.0], 140 mM KCl, 1.8 mM MgCl_2_, 0.1% Nonidet P-40 [NP-40], 0.02 mg/ml heparin) and resuspended in 5 ml of buffer B (buffer A plus 0.5 mM dithiothreitol [DTT], 1 mM phenylmethylsulfonylfluoride, 0.5 μg/ml leupeptin, 0.8 μg/ml pepstatin, 20 U/ml DNase I, 100 U/ml RNasin [Promega, Madison, Wisconsin, United States], and 0.2 mg/ml heparin). Cells were broken mechanically with glass beads, and extracts were incubated with 400-μl slurry (50% [v/v]) IgG–agarose beads (Sigma, St. Louis, Missouri, United States) for 2 h at 4°C. The beads were washed four times for 15 min at 4°C with buffer C (20 mM Tris–HCl [pH 8.0], 140 mM KCl, 1.8 mM MgCl_2_, 0.5 mM DTT, 0.01% NP-40, 10 U/ml RNasin). Puf proteins were released from the beads by incubation with 80 U of TEV protease (Invitrogen) for 2 h at 15°C. RNA was isolated from the TEV eluates, which corresponds to the purified fraction and from extracts (input) by extraction with phenol/chloroform and isopropanol precipitation.

#### Microarray analysis and data selection

Equal amounts of a pool of five synthetically prepared Bacillus subtilis RNAs were added to each RNA sample prior to labeling and served as a control for the labeling procedure (Wang et al. 2002b). Total RNA (3 μg) derived from the extract and 300 ng of affinity-isolated RNA (or up to 40% of isolated RNA) were labeled with Cy3 and Cy5 fluorescent dyes, respectively, following cDNA synthesis with amino-allyl dUTP in addition to the four natural dNTPs using a 1:1 mixture of oligo(dT) and random nonamer primers. The Cy3- and Cy5-labeled cDNA samples were mixed and competitively hybridized to DNA microarrays representing all S. cerevisiae ORFs, introns, and the mitochondrial genome (see http://brownlab.stanford.edu/protocols.html). Microarrays were scanned with an Axon Instruments (Foster City, California, United States) Scanner 4000. Scanning parameters were adjusted to give similar fluorescent intensities for B. subtilis spots in both channels. Data were collected with the GENEPIX 3.0 Program (Axon Instruments), and spots with abnormal morphology were excluded from further analysis. Arrays were computer normalized by the Stanford Microarray Database (SMD) (Gollub et al. 2003). Log_2_ median ratios were retrieved from SMD and exported into Microsoft (Redmond, Washington, United States) Excel after filtering for regression correlation of greater than 0.6 (filters for large variations in the ratios of pixels within each spot), CH1I/CH1B of greater than 1.8 (signal over background in the channel measuring total RNA from extract), and CH2I/CH2B of greater than 1.0 (affinity-isolated RNA signal greater than background) and for data from at least two independent measurements. Average log_2_ ratios were calculated for each gene across the four independent experiments performed for each Puf protein (microarrays and raw data can be downloaded from our supporting Web sites [http://microarray-pubs.stanford.edu/yeast_puf/ and http://genome-www5.stanford.MicroArray/SMD/]). Genes for which the enrichment ratios were at least two standard deviations above the median across all genes were selected. A total of 923 genes were selected in this way. To eliminate nonspecifically enriched RNAs from this gene list, the results from the affinity enrichments for each of the Puf proteins and the data obtained from four independent mock affinity enrichments were clustered by the Pearson correlation algorithm (Eisen et al. 1998). Transcripts of 84 genes were enriched beyond the two standard deviation threshold in all the Puf affinity isolations as well as in the mock procedure. These were presumed to represent RNAs whose enrichment was unrelated to specific interactions with Puf proteins and therefore were excluded from further analysis. Among the finally selected target mRNAs (see Tables S3–S7), most were represented in the four independent measurements: *PUF1*, 98%; *PUF2*, 97%; *PUF3*, 82%; *PUF4*, 93%; *PUF5*, 97%.

#### Gene expression profiling

*puf3* mutant and wild-type cells were cultured in minimal media supplemented with 3% glycerol and harvested during exponential growth (OD_600_ = 0.5). Total RNA (8 μg) isolated from wild-type and mutant cells were used to prepare Cy3 and Cy5 fluorescently labeled cDNA as described above, except that only an oligo(dT) primer was used. The two differentially labeled cDNAs were mixed together and hybridized to yeast DNA microarrays. Arrays were scanned and the data were collected, entered into SMD, and computer normalized (Gollub et al. 2003). Log_2_ median ratios were retrieved from SMD after filtering for regression correlation of greater than 0.6 and signal over background of greater than 1.5. Results from three independent experiments were averaged for this analysis (raw data can be retrieved from our Web site).

#### Motif searches

As the exact 5′- and 3′-UTR lengths are unknown for most of the Puf target mRNAs, we used the estimated average lengths from yeast (Mignone et al. 2002). Hence, the coding 237 nucleotides of predicted 3′-UTR and 134 nucleotides of predicted 5′-UTR sequences were retrieved from SGD for the Puf target genes. The sequences were searched for motifs in the sense strand with the program MEME under the proposed default settings (http://meme.sdsc.edu/meme/website/intro.html) (Bailey and Elkan 1994) (see Tables S9–S11). The number and location of consensus motifs in the S. cerevisiae genome was obtained by searching “Pattern Match” in the SGD (Issel-Tarver et al. 2002). Thereby, nucleotides that were at least 19% conserved among the MEME selected sequences were used to compile the Consensus Motif that was searched for.

#### Three-hybrid assays

Three-hybrid assays were performed as described elsewhere (Bernstein et al. 2002).

#### Immunofluorescence

Immunofluorescence was performed as described at http://www.med.unc.edu/%7Ehdohlman/IF.html. Fixed and permeabilized cells were treated with 5 μg/ml purified rabbit immunoglobulin (Sigma) for 1 h at room temperature. After washing, cells were incubated with Cy3 goat anti-rabbit antibodies (1:400). Images were obtained on a Zeiss (Oberkochen, Germany) Axioplan-2 microscope using an Axiocam HRC camera.

## Supporting Information

Full microarray results and other supporting information can be viewed at http://microarray-pubs.stanford.edu/yeast_puf/ and at http://genome-www5.stanford.MicroArray/SMD/.

Figure S1 Distribution of Average Cy5/Cy3 Fluorescence Ratios from Quadruplicate Microarray Hybridizations Analyzing mRNA Targets for Puf1p, Puf2p, Puf4p, and Puf5p See Figure 3A for Puf3p.
(167 KB EPS).

Figure S2 Examples of Groups of mRNAs Associated with the Same Puf Protein and Encoding Related Proteins (A) Puf2p-bound mRNAs encode diverse proteins involved in regulation of ATP-dependent proton transport. *PMA1* and *PMA2* encode plasma membrane proteins that comprise the major ATP-dependent proton transporters and regulate cellular pH levels. Pmp1p, Pmp2p, and Pmp3p are small isoproteolipids, which are present in a physical complex with Pma1p and act as regulators of its activity upon stress conditions (Navarre et al. 1994). Hrk1p is a protein histidine kinase, which activates Pma1p in response to glucose (Goossens et al. 2000). Ast1p is implicated in proper delivery of Pma1p to plasma membranes (Bagnat et al. 2001).
(B) Puf4p-bound mRNAs encode the nucleolar GAR proteins (blue), members of the H/ACA core complex (boxed), and Hmt1p, a dimethylase acting on GAR proteins. Nop1p performs 2′-*O*-ribose methylation of pre-rRNA, a process guided by small nucleolar RNAs (snoRNAs) of the box C/D family. Cbf5p catalyzes pseudouridine formation with box H/ACA snoRNAs, and three of the four components of the H/ACA core complex were Puf4p-associated (Cbf5, Gar1, and Nhp2 [Henras et al. 1998]; no data were obtained for the fourth component, Nop10, shown in gray). All transcripts encoding nucleolar proteins of the GAR repeats family (Gar1p, Sbp1p, Nop1p, Nsr1p) were Puf4p-bound. The GAR domain is dimethylated at arginine residues. Remarkably, several mRNAs coding for S-adenosylmethionine-dependent methyltransferases were Puf4p-bound including Hmt1p, the major protein arginine-methyltransferase in yeast (Gary et al. 1996). Hmt1p has recently been shown to dimethylate arginines of the proteins Gar1p, Nop1p, and Nsr1p (Xu et al. 2003).
(38 KB EPS).

Figure S3 Phenotypic Analysis of *puf3*Δ Cells Serial dilutions (1:10) of cells were spotted on plates supplemented with the indicated media. Plates were incubated for 3 d at 30°C. Abbreviations: YPD, yeast–peptone–dextrose; YPGE, yeast–peptone–3% glycerol–2% ethanol; SC, synthetic complete.
(264 KB PDF).

Table S1 Number of mRNA Targets Shared between Puf Proteins (15 KB XLS).

Table S2 Protein Copy Number Determination of Puf Proteins Cells were grown to mid-log phase in YPAD medium and the number of cells was counted. Whole-cell extracts were prepared as described previously (Hoffman et al. 2002). In brief, cells were resuspended in 1× SDS-PAGE sample buffer, incubated at 100°C for 10 min, and vortexed for 2 min with glass beads. After a short centrifugation, eight dilutions of cell extracts and protein A (Amersham, Little Chalfont, United Kingdom), which served as a reference standard, were spotted on a nitrocellulose filter. Expression of IgG-binding domains was monitored with rabbit peroxidase–anti-peroxidase soluble complex at 1:5,000 (Sigma). Chemiluminescence was measured with a Typhoon 8600 Imager (Molecular Dynamics, Sunnyvale, California, United States) and quantified with the ImageQuant 5.2 software. Averaged numbers from two independent measurements were used for calculations. The total number of mRNA copies in the pool associated with each Puf protein was estimated as follows: copy numbers for individual mRNAs were retrieved from two independent genome-wide measurements (Holstege et al. 1998; Wang et al. 2002b). For genes with no data, we added the median value for copy numbers of all mRNAs in the respective pool.
(30 KB XLS).

Table S3 List of Puf1p Target mRNAs Columns indicate the following (from left to right): ORF; gene name; GO annotations; classification of gene products (soluble/membrane-associated); average log_2_ ratios of enrichment across four independent Puf affinity isolations; standard deviations; association of mRNA with other Puf proteins; mRNA copy numbers.
(28 KB XLS).

Table S4 List of Puf2p Target mRNAs Notations are as in Table S3.
(52 KB XLS).

Table S5 List of Puf3p Target RNAs Columns indicate the following (from left to right): ORF; gene name; GO annotations; classification of gene products (soluble/membrane-associated); average log_2_ ratios of enrichment across four independent Puf affinity isolations; standard deviations; association of mRNA with other Puf proteins; location of consensus motif identified by MEME; mRNA copy numbers.
(70 KB XLS).

Table S6 List of Puf4p Target mRNAs Notations are as in Table S5.
(61 KB XLS).

Table S7 List of Puf5p Target mRNAs Notations are as in Table S5.
(64 KB XLS).

Table S8 Significant Shared GO Annotations among Puf mRNA Targets Only annotations with *p* values of less than 0.001 are indicated. GO annotations were retrieved from the SGD with GO Finder (http://db.yeastgenome.org/cgi-bin/SGD/GO/goTermFinder) on May 21, 2003. Respective *p* values are indicated in a column next to the names of the GO term.
(30 KB XLS).

Table S9 Results of MEME Motif Searches: Motifs among Puf3p mRNA Targets (63 KB XLS).

Table S10 Results of MEME Motif Searches: Motifs among Puf4p mRNA Targets (55 KB XLS).

Table S11 Results of MEME Motif Searches: Motifs among Puf5p mRNA Targets (34 KB XLS).

### Accession Numbers

All accession numbers for human, *Drosophila*, or C. elegans proteins are from the SwissProt database (http://www.ebi.ac.uk/swissprot/): CPEB (Q18317), GLD1 (Q17339), DAZL (Q92904), FBF-1 (Q9N5M6), FEM3 (P34691), IRP (P21399), NANOS (P25724), *Drosophila* PUMILIO (P25822), human PUMILIO-1 (Q14671), human PUMILIO-2 (Q9HAN2), and SLBP (P97330).

The accession numbers for S. cerevisiae genes are from SGD (http://genome-www.stanford.edu/Saccharomyces/) (ORF/SGD identification number): *ADA2* (YDR448W/S0002856), *AME1* (YBR211C/S0000415), *APS3* YJL024C/S0003561), *AST1* (YBL069W/S0000165), *BBP1* (YPL255W/S0006176), *BDF1* (YLR399C/S0004391), *BDF2* (YDL070W/S0002228), *BOI2* (YER114C/S0000916), *BSP1* (YPR171W/S0006375), *BUB1* (YGR188C/S0003420), *BUD9* (YGR041W/S0003273), *CBF5* (YLR175W/S0004165), *CDC31* (YOR257W/S0005783), *CNM67* (YNL225C/S0005169), *COX17* (YLL009C/S0003932), *DAD2* (YKR083C/S0001791), *DHH1* (YDL160C/S0002319), *ELM1* (YKL048C/S0001531), *FPS1* (YLL043W/S0003966), *GAR1* (YHR089C/S0001131), *GIC1* (YHR061C/S0001103), *HDA1* (YNL021W/S0004966), *HFI1* (YPL254W/S0006175), *HMT1* (YBR034C/S0000238), *HOS1* (YPR068C/S0006272), *HOS3* (YPL116W/S0006037), *HST1* (YOL068C/S0005429), *HTA1* (YDR225W/S0002633), *IFM1* (YOL023W/S0005383), *KAR1* (YNL188W/S0005132), *KAR9* (YPL269W/S0006190), *KHD1* (YBL032W/S0000128), *MAS6* (YNR017W/S0005300), *MEX67* (YPL169C/S0006090), *MUP3* (YHL036W/S0001028), *NCE101* (YJL205C/S0003742), *NCE102* (YPR149W/S0006353), *NHP2* (YDL208W/S0002367), *NOP1* (YDL014W/S0002172), *NSR1* (YGR159C/S0003391), *NUF2* (YOL069W/S0005430), *PDR16* (YNL231C/S0005175), *PMA1* (YGL008C/S0002976), *PUF1* (YJR091C/S0003851), *PUF2* (YPR042C/S0006246), *PUF3* (YLL013C/S0003936), *PUF4* (YGL014W/S0002982), *PUF5* (YGL178W/S0003146), *RAX2* (YLR084C/S0004074), *RSC1* (YGR056W/S0003288), *RSC2* (YLR357W/S0004349), *RSC4* (YKR008W/S0001716), *SBP1* (YHL034C/S0001026), *SCP160* (YJL080C/S0003616), *SFK1* (YKL051W/S0001534), *SFL1* (YOR140W/S0005666), *SHE2* (YKL130C/S0001613), *SIN3* (YOL004W/S0005364), *SNC2* (YOR327C/S0005854), *SNT1* (YCR033W/S0000629), *SPC19* (YDR201W/S0002609), *SPC42* (YKL042W/S0001525), *SPT7* (YBR081C/S0000285), *SPT8* (YLR055C/S0004045), *SSO2* (YMR183C/S0004795), *STE7* (YDL159W/S0002318), *SUR7* (YML052W/S0004516), *TPO1* (YLL028W/S0003951), *TPO2* (YGR138C/S0003370), *TPO3* (YPR156C/S0006360), *VPS72* (YDR485C/S0002893), *YIP1* (YGR172C/S0003404), *YKL091c* (YKL091C/S0001574), *YPR157w* (YPR157W/S0006361), and *YRA1* (YDR381W/S0002789).

## Abbreviations

DAZ: deleted in azoospermia
DTT: dithiothreitol
FBF: Fem-3-binding factor
GAR: glycine/arginine-rich
GO: Gene Ontology
HDAC: histone deacetylase
ORF: open reading frame
IRP: iron regulatory protein
MEME: multiple expectation maximization for motif elicitation
NP-40: Nonidet P-40
Puf: Pumilio-FBF
Pum-HD: Pumilio homology domain
RBP: RNA-binding protein
rRNA: ribosomal RNA
SAGA: Spt–Ada–Gcn5–acetyltransferase
SGD: *Saccharomyces* Genome Database
SLBP: stem–loop binding protein
SMD: Stanford Microarray Database
snoRNA: small nucleolar RNA
STAR: signal transduction and activation of RNA
TAP: tandem-affinity purification
TEV: tobacco etch virus
UTR: untranslated region
YPD: Yeast Proteome Database
YPD: yeast–peptone–dextrose
YPGE: yeast–peptone–3% glycerol–2% ethanol
