# Supplementary figures and images for: Extensive Association of Functionally and Cytotopically Related mRNAs with Puf Family RNA-Binding Proteins in Yeast
Source: PLoS Biol. 2004 Mar 16;2(3):e79. doi: 10.1371/journal.pbio.0020079 (PMC368173; doi:10.1371/journal.pbio.0020079)

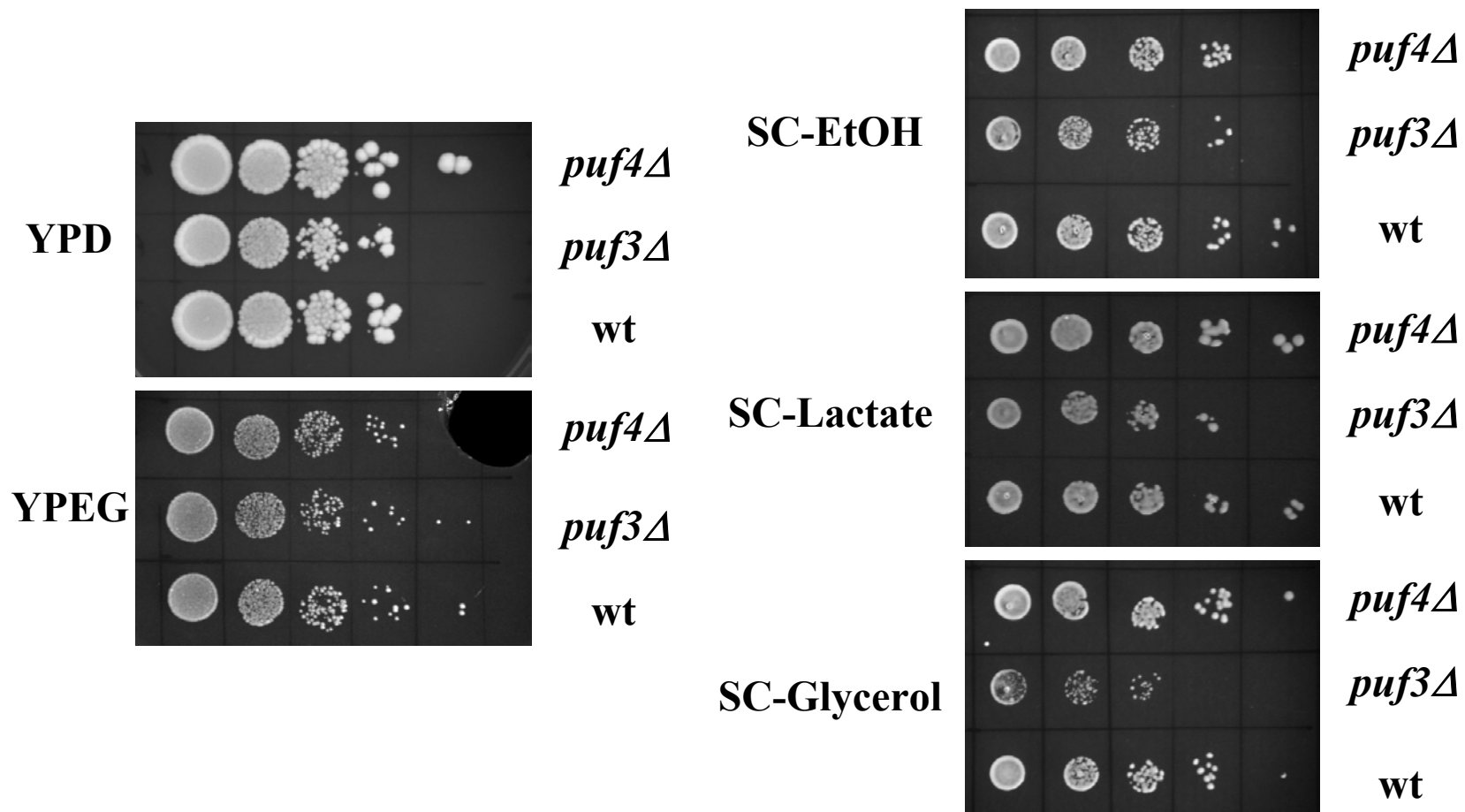

Supplement: Figure S3 — Serial dilutions (1:10) of cells were spotted on plates supplemented with the indicated media. Plates were incubated for 3 d at 30°C. Abbreviations: YPD, yeast–peptone–dextrose; YPGE, yeast–peptone–3% glycerol–2% ethanol; SC, synthetic complete. (264 KB PDF). [file pbio.0020079.sg003.pdf]
